# Modeling Late-Summer Distribution of Golden Eagles (*Aquila chrysaetos*) in the Western United States

**DOI:** 10.1371/journal.pone.0159271

**Published:** 2016-08-24

**Authors:** Ryan M. Nielson, Robert K. Murphy, Brian A. Millsap, William H. Howe, Grant Gardner

**Affiliations:** 1 Western EcoSystems Technology, Inc., Cheyenne, Wyoming, United States of America; 2 United States Fish and Wildlife Service, Division of Migratory Birds, Albuquerque, New Mexico, United States of America; University of Lleida, SPAIN

## Abstract

Increasing development across the western United States (USA) elevates concerns about effects on wildlife resources; the golden eagle (*Aquila chrysaetos*) is of special concern in this regard. Knowledge of golden eagle abundance and distribution across the western USA must be improved to help identify and conserve areas of major importance to the species. We used distance sampling and visual mark-recapture procedures to estimate golden eagle abundance from aerial line-transect surveys conducted across four Bird Conservation Regions in the western USA between 15 August and 15 September in 2006–2010, 2012, and 2013. To assess golden eagle-habitat relationships at this scale, we modeled counts of golden eagles seen during surveys in 2006–2010, adjusted for probability of detection, and used land cover and other environmental factors as predictor variables within 20-km^2^ sampling units randomly selected from survey transects. We found evidence of positive relationships between intensity of use by golden eagles and elevation, solar radiation, and mean wind speed, and of negative relationships with the proportion of landscape classified as forest or as developed. The model accurately predicted habitat use observed during surveys conducted in 2012 and 2013. We used the model to construct a map predicting intensity of use by golden eagles during late summer across our ~2 million-km^2^ study area. The map can be used to help prioritize landscapes for conservation efforts, identify areas where mitigation efforts may be most effective, and identify regions for additional research and monitoring. In addition, our map can be used to develop region-specific (e.g., state-level) density estimates based on the latest information on golden eagle abundance from a late-summer survey and aid designation of geographic management units for the species.

## Introduction

Populations of the golden eagle (*Aquila chrysaetos*) appear stable in western regions of the USA [1,2]. This status may be short-lived, however, because the eagle’s reproductive potential is low, i.e., it is a *K*-strategist species [3,4], and additive mortality posed by increasing anthropogenic threats could trigger declines. Examples of direct threats include mortality due to electrocution on power lines, collisions with vehicles and human-created structures such as wind turbines, and poisoning [5,6]. Moreover, quantity and quality of the eagle’s habitat in the western USA likely is declining, with concurrent decreases in prey availability. Decreased habitat quantity includes conversion of landscapes preferred by the eagle to land use dominated by urbanization, agriculture, or energy development [5]. An example of diminished quality of golden eagle habitat in much of the western USA is invasion by introduced plant species into native sagebrush (*Artemisia* spp.) shrubsteppe communities [7], leading to less favorable conditions for key prey species such as black-tailed jackrabbits (*Lepus californicus* [8]).

Basic models for estimating distribution and habitat use of golden eagles at regional scales [9] are urgently needed to conserve the species in the western USA. Models that predict intensity of use by golden eagles based on landscape characteristics could be used to illustrate the eagle’s distribution. This would help resource managers first identify landscapes of potential significance to the species, then prioritize evaluations at smaller scales to identify potential threats and management opportunities [10,11]. Moreover, models that predict habitat use based on landscape characteristics are needed to inform compensatory mitigation for offsetting incidental take of golden eagles currently allowed on a limited basis under permit from the United States Fish and Wildlife Service (Service) [12]. To predict the impact of such take for a given action (e.g., potential blade-strike mortality at a wind energy project), the Service first estimates the number of golden eagles at a local scale encompassing the site where take may occur [13]. This estimate is coarse, owing to an assumption that golden eagles are uniformly distributed across ca. 100,000-km^2^ landscapes often having diverse habitat features. The estimates could be much improved by using habitat-based distribution models to stratify density estimates. Last, habitat-based distribution models are needed to optimally allocate sampling effort in demographic surveys of golden eagles, as the surveys can otherwise be prohibitively expensive. Other than a recent model for predicting nest site locations of golden eagles in Wyoming, USA [14], no predictive models have been published for golden eagles across the western USA. In North America, this wide-ranging species uses a variety of land cover and land use types, and environmental factors such as terrain ruggedness and elevation strongly influence its occurrence in a given locale [5]. Resource selection functions (RSFs) could be used to help construct models for predicting a species’ distribution. Typically, RSFs are estimated by comparing a set of ‘used’ locations to those from an ‘available’ set. This approach treats the response as binary and does not account for intensity of use among sampled habitat units [15]. Here, we use a count-based regression approach to estimate a RSF that models intensity of use by golden eagles during late summer. Because our study area encompassed most of the breeding range of golden eagles in the western USA, our RSF represents an investigation similar to first- and second-order analysis of habitat use [16].

**Fig 1:**
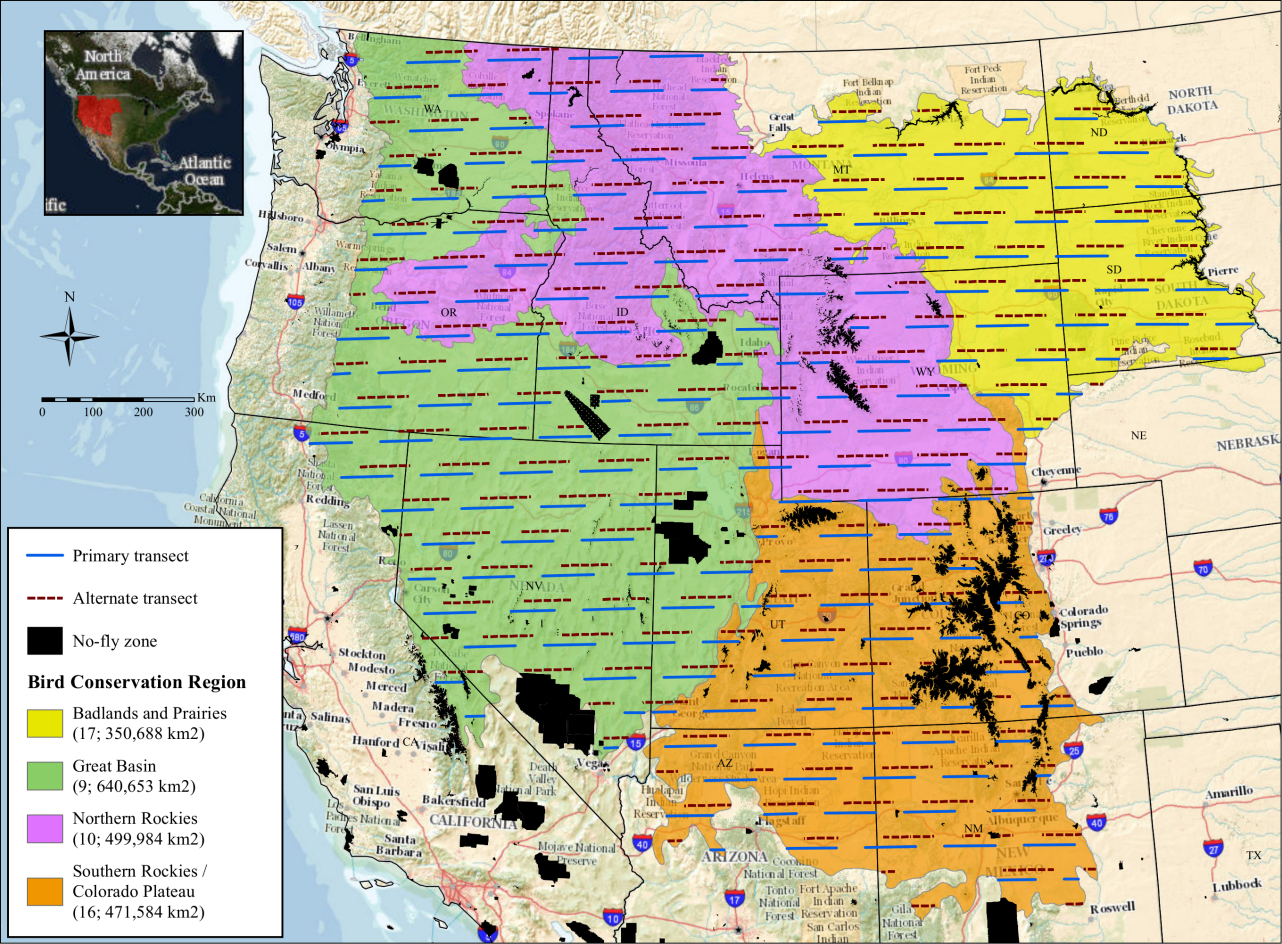
Study area for the annual late-summer survey of golden eagles across the western USA. In every year during 2006–2013 we attempted to survey all primary transects, but alternates were flown when portions of primary transects could not be flown due to forest fires or weather events. Image background is from The National Map (United States Geological Survey).

As part of long-term monitoring of golden eagle abundance across four major Bird Conservation Regions (BCRs [17]) in the western USA, annual surveys of the species were conducted along roughly 17,500 km of aerial transects during late summer in 2006–2010, 2012, and 2013, using line-transect distance sampling ([2]; Fig 1). The primary objective for this western golden eagle survey (WGES) was to provide the Service with accurate estimates of abundance and long-term trends in population size. Here, we use data from the WGES to model counts of golden eagle observations across the four BCRs based on land cover and other environmental factors. Our objective was to identify which of these landscape-level, environmental factors are linked with coarse-scale habitat selection by golden eagles during late summer and use those factors to predict the distribution of golden eagles across the four western USA BCRs during this season. We sought to produce a final model and distribution map to help prioritize areas for managing and conserving the species, and to develop a robust framework for estimating golden eagle densities in many sub-regions of the western USA. Our approach could be broadly applied to further the conservation of other species of wildlife.

## Materials and Methods

### Study Area

The study area consisted of BCRs 9 (Great Basin), 10 (Northern Rockies), 16 (Southern Rockies and Colorado Plateau), and 17 (Badlands and Prairies) within the USA (Fig 1). These regions collectively covered about 80% of the golden eagle’s range in the coterminous western USA [12]. Habitat types across the BCRs ranged mainly from low-elevation (600–1,700 m), semi-arid sagebrush and grassland basins to high-elevation (2,600–3,200 m) coniferous forests and montane meadows. Department of Defense lands, urban areas, bodies of water greater than 30,000 ha, and terrain above 3,048 m comprised 6.7% of the total study area; we did not survey these zones due to safety reasons or restricted access. Excluding these zones, the total area in our sample frame for 2006–2010, 2012 and 2013, encompassing all four BCRs, was 1,962,909 km^2^ (Fig 1).

### Surveys

Our field methodology is detailed in Nielson et al. [2] and summarized here. During 2006–2013, we annually conducted aerial distance sampling [18] surveys from fixed-wing aircraft from 15 August to 15 September, after all juvenile golden eagles were expected to have fledged and before most golden eagles began their fall migrations [5,19]. We established transects by randomly overlaying the study area with two systematic sets of 100-km long, east-west transects (systematic sample with a random start; Fig 1). The first systematic set contained the primary transects. The second set contained alternate transects to be surveyed if a primary transect could not be flown due to low clouds, storms, or wildfires. Primary and alternate transects were spaced roughly 60-km east-to-west and north-to-south. After removing portions of transects extending outside the study area or over zones that were avoided due to safety reasons or restricted access, each systematic set comprised about 17,500 km of transects. We did not survey BCR 17 in 2011 so that we could investigate effects of increased survey effort in BCR 16, so we did not include data from 2011 in this analysis.

We used two crews to complete surveys each year. With few exceptions (Table 1), each crew consisted of three observers–two seated side-by-side in the back seat and the third in the front-right seat of the aircraft. Surveys began at sunrise and ended by 1300 hours. Early morning transects usually were flown from east to west to provide the best possible lighting for detecting golden eagles. Surveys were flown at about 160 km hr^-1^. We flew at 107 m above ground level (AGL) over open, level to rolling terrain and, for safety reasons, at 150 m AGL over forested, rugged, or mountainous terrain. We used a GPS to record the location of each golden eagle group where first observed, regardless of age, whether flying or perched, and whether resident or non-resident.

**Table 1:** Estimated probability of detection (SEs in parentheses) of golden eagle groups from each side of the aircraft based on surveys conducted 2006–2013 [2]. Three observers (back-left, and front-right and back-right) were present on surveys with exception of 68 transects in 2008, where only a back-left observer and a front-right observer were available.

	Observer Position in Aircraft		
Observation Type	Back-Left	Right (front and back)	Front-Right
Perched: 107 m AGL^a^	0.467 (0.026)	0.588 (0.029)	0.456 (0.026)
Perched: 150 m AGL^b^	0.280 (0.047)	0.396 (0.051)	0.280 (0.045)
Flying	0.380 (0.066)	0.472 (0.058)	0.301 (0.038)

^a^Birds seen while perched when the aircraft was flying 107 m Above Ground Level (AGL)

^b^Birds seen while perched when the aircraft was flying 150 m Above Ground Level (AGL)

The survey of golden eagles upon which this paper was based was non-interventive and required no special permits; eagles were observed from aircraft at distances far greater than distances of aircraft they react to [20], and no eagles were captured or handled. A visual mark-recapture procedure was used in the study, but this method [2] was also non-interventive. Potential observer effects (e.g., some better than others) was controlled by yearly pre-survey training, using only experienced golden eagle biologists, rotating observers within the aircraft on a daily basis, and rotating north and south crews.

Due to the sensitive nature of GPS locations of golden eagles used in this study the Service must use a risk-averse approach in granting open access to the GPS location data. Many of the data are associated with golden eagle nest sites, some of which are on lands owned by Native American Tribes and private individuals, and thus cannot be divulged to the public without express permission directly from these parties. Moreover, the species receives special protections under the federal Bald and Golden Eagle Protection Act, including prohibition of take due to disturbance at nest sites and other critical use areas, which may be discerned by the GPS location data used in this study. Although the Service is unwilling to allow open access to GPS location data used in this study, individual requests for the data can be sent to the Service (Robert K. Murphy; robert_murphy@fws.gov) and will be addressed on a case-by-case basis.

## Statistical Analysis

Our approach to developing a model of late-summer habitat use by golden eagles (hereafter, the model) consisted of four steps in which we adjusted raw counts of golden eagles based on estimated probabilities of detection, took a random sample of points along primary transects, assigned the adjusted golden eagle counts to those sampling points, and then modeled the distribution of adjusted counts. First, to estimate the number of golden eagles actually present during the surveys, we inflated each golden eagle group (≥1 individual) observed during 2006–2010 by the estimated average probability of detection for that type of observation (e.g., whether the eagle was flying or perched; Table 1). Average probabilities of detection were estimated as a function of distance from the line-transect, or minimum available sighting distance, whether the observation was on the left or right side of the aircraft (i.e., whether one or two observers were present), observation type, and AGL of the aircraft when the observation was recorded. We estimated probabilities by using all survey data collected 2006–2013. Methods and assumptions behind the estimated probabilities of detection have been described in detail [2].

**Fig 2:**
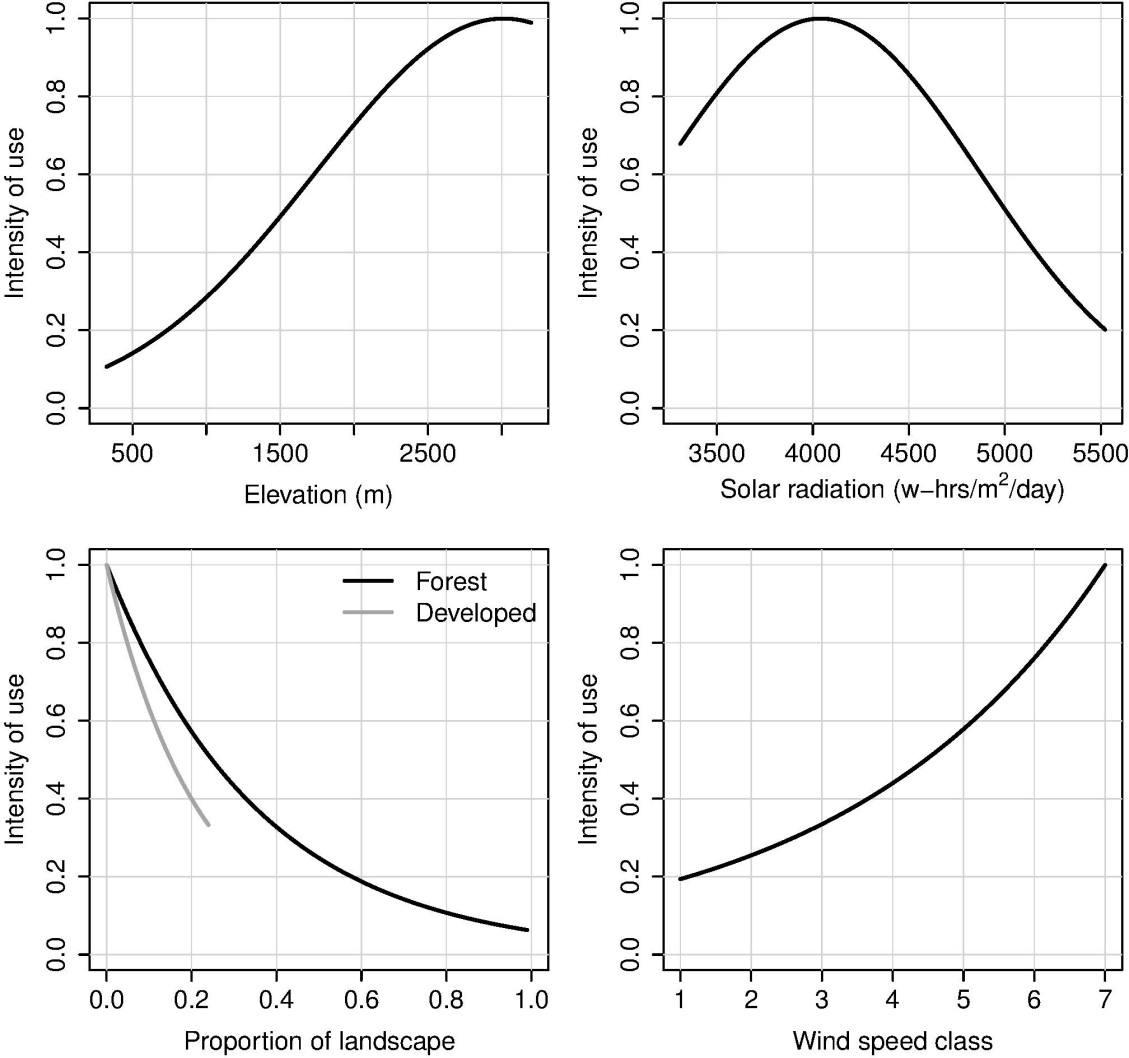
Predicted intensity of use by golden eagles as a function of covariates in the final model. Covariate values not represented in each graph were held constant at their median values, and final estimates were scaled so the maximum value was 1.0.

Second, we took a simple random sample with replacement of 2,000 points along primary transects to ensure independence of the points [21]. We centered 2-km x 10-km rectangular cells on the sampled points and considered these cells our sampling units. We discarded sampling units that extended beyond transects on their eastern or western ends. Sampling units were 2 km long on their north-south borders because the WGES focused on detecting golden eagles out to 1,000 m from transect lines (Fig 1; [2]). Sampling units were 10 km long for several reasons: 1) golden eagle observations were collected via a study designed for inference across large geographic regions (Fig 1), 2) many measures of terrain and habitat characteristics (described below) available across our inference space were not suited for finer-scale analysis, and 3) counts of golden eagles along smaller stretches of transects would have been dominated by zero values, which would have complicated modeling (described below).

For the third step in developing our model, we summed the inflated counts of golden eagles across years 2006–2010 within each sampling unit. For example, suppose two groups of golden eagles were observed in sampling unit i in 2006, and group sizes were one and two, respectively. The first group was seen flying by the back-left observer, and the second group was seen perched by the right side observer(s) while surveying from 107 m AGL. Only one golden eagle was seen in 2007–2010; it was perched, and the right side observer(s) detected it from a survey height of 107 m AGL. Using the detection probabilities in Table 1, the inflated count of golden eagles observed within sampling unit i 2006–2010 was counti = (1/0.380) + (2/0.588) + (1/0.588) = 7.734. We rounded sums of adjusted counts to represent “pseudocounts that can be analyzed using a count regression” [22]. We chose this approach because the distribution of responses appeared similar to typical count data–highly skewed with a small mean and variance and several zero counts–except our values were continuous due to adjustments for probability of detection.

As the fourth and final step in our analysis, we used a generalized linear modeling approach [23] to relate sums of adjusted counts to landscape-level covariates describing each sampled cell. Count data typically are modeled by using negative binomial (NB) regression [23–25,19–21]. Frequency of use in a sample of ‘habitat’ units can be regressed against characteristics of those units (covariates). This contemporary RSF approach models intensity of use rather than the binary response used in traditional RSFs that compare a discrete set of “used” locations to those from a sample of “available” locations [9,15]. The NB regression technique has been successfully used to model intensity of use for other species [15,26–29]. We modeled the rounded, inflated count of golden eagles in sampling unit i using a NB regression model [18]:

$$\ln\left[E\left(rounded\left\{count_{i}\right\}\right)\right]=\ln\left[n.years_{i}\right]+\beta_{0}+\beta_{1}x_{1i}+\beta_{2}x_{2i}+\ldots +\beta_{p}x_{pi},$$ (1)

where ln was the natural logarithm (base e); counti was the sum of counts of golden eagles within sampling unit i, adjusted by probabilities of detection; n.yearsi was the number of years sampling unit i was surveyed during 2006–2010 (usually five); *β*_0_ was an intercept term; *β*_1_,*β*_2_,…,*β*_*p*_, were coefficients to be estimated for landscape-level covariates; *x*_1*i*_, …, *x*_*pi*_ were values of p covariates measured on sampling unit I; and E[.] denoted the expected value. Inclusion of the offset term ln[n.yearsi] in Eq (1) simply scaled the response according to effort, i.e., the number of years the sampling unit was surveyed. We were more likely to discontinue higher elevation and mountainous sections of transects due to low clouds, storms, and wildfires. If this change in effort was ignored, the final model could lead to erroneous conclusions.

For landscape-level, environmental factors, we considered 18 covariates (Table 2) that represent facets of habitat generally thought to be associated, either positively (e.g., terrain ruggedness, land cover comprised by shrub types) or negatively (e.g., human population density; land cover comprised by agriculture), with golden eagle abundance and distribution in various areas or regions of North America; literature describing these associations is synthesized in Kochert et al. [5]. These 18 represented the total list of *a priori* covariates we were able to obtain for the entire study area. We calculated median values of elevation and slope within each sampling unit based on the U.S. Geological Survey (USGS) National Elevation Dataset (30-m resolution). We also identified the predominant aspect (N, S, E, or W) of each sampling unit, and calculated the median value of terrain ruggedness within each sampling unit. We used the vector ruggedness measure presented in Sappington et al. [30] as our measure of terrain ruggedness, which is less correlated with slope compared to other ruggedness indices. We calculated the median value of the 2008 Normalized Digital Vegetation Index (NDVI; 1.1-km resolution; EROS Data Center, USGS) for each sampling unit. Measures of NDVI represented the amount of visible light absorbed by plants as well as the amount of near-infrared light that plants reflected [31]; we used NDVI as a gross indicator of spatial variation in plant productivity, which may influence levels of prey biomass available to eagles. Median human population density, distance to nearest primary road, and distance to nearest secondary road were obtained from the U.S. Census Topologically Integrated Geographic Encoding and Referencing Product. Distances to primary or secondary roads (50-m resolution) were from the center of each sampling unit because a median value would be highly correlated with the center point value. We obtained a solar radiation layer and a wind speed class layer from the National Renewable Energy Laboratory. The solar radiation layer (resolution of 0.1 degree latitude and longitude = ~10 km) contained the mean solar radiation for August 2008 (median modeling year); we considered solar radiation as a gross indicator of aridity across the study area, which may affect local to regional abundances of eagles by influencing thermoregulation among individuals, behavior (e.g., estivation) and thus availability of prey, and plant productivity. The wind speed class layer had a resolution of 0.25 degrees latitude by 0.33 degrees longitude. This covariate represented seven wind speed classes based on mean wind speeds at 50 m AGL: 0, 5.6, 6.4, 7.0, 7.5, 8.0, 8.8, and 11.9 m s^-1^. Median values of both solar radiation and wind speed class were calculated for each sampling unit. For every sampling unit, we used the 2006 National Land Cover Database (NLCD; 30-m resolution [32]) to identify shrub/scrub, grassland/herbaceous, crop/hay, developed, forest, barren, wetland, or water/ice/snow land cover classes then calculated the proportion that each class comprised (Table 3).

**Table 2:** Covariates considered for the model of late-summer habitat use by golden eagles.

Covariate Name	Description
Elevation	Median elevation (m)
Slope	Median slope (degrees)
Aspect	Predominant aspect (N, S, E, W)
NDVI	Median Normalized Digital Vegetation Index (2008)
Solar radiation	Median solar radiation (watt-hours m^2^–^1^ day^-1^)
Population density	Median human population density (number km^**2–1**^)
Terrain ruggedness	Median terrain ruggedness (0–1 scale)
Road1	Distance (km) to nearest primary road
Road2	Distance (km) to nearest secondary road
Wind speed class	Median wind speed class, classes 1 through 7
Shrub/scrub	Proportion of shrub/scrub (NLCD 2006)
Grassland/herbaceous	Proportion of grassland/herbaceous (NLCD 2006)
Crop/hay	Proportion of cultivated crops or pasture/hay (NLCD 2006)
Developed	Proportion of developed (NLCD 2006)
Forest	Proportion of forest (NLCD 2006)
Barren	Proportion of rock/sand/clay (NLCD 2006)
Wetland	Proportion of woody or emergent herbaceous wetlands (NLCD 2006)
Water/ice/snow	Proportion of open water and/or perennial ice/snow (NLCD 2006)

**Table 3:** Land cover covariates and their definitions considered for the model of late-summer habitat use by golden eagles. The proportion comprised by each covariate was determined for every 2- × 10-km sampling unit. Modified from land cover classes and their definitions in the 2006 National Land Cover Database [32].

Covariate Name	Definition
Shrubland	Areas with more than 20% cover comprised by natural or semi-natural woody vegetation with aerial stems less than 6 m tall; includes trees or shrubs that are small or stunted because of edaphic conditions
Grassland/herbaceous	Areas with more than 80% graminoid or other herbaceous vegetation cover and not subject to intensive management such as tilling but may be used for grazing
Crop/hay	Areas with 75–100% herbaceous vegetation cover that has been planted or is intensively managed for the production of food, feed, or fiber; or is maintained in developed settings for specific purposes
Developed	Areas with more than 30% cover comprised by constructed materials, e.g. asphalt, concrete, buildings
Forest	Areas with 25–100% natural or semi-natural woody vegetation cover more than 6 m tall
Barren	Bare rock, gravel, sand, silt, clay, or other earthen material, with little or no green vegetation present
Wetland	Areas where the soil or substrate is periodically saturated with or covered with water
Water/ice/snow	Open water with less than 25% cover of vegetation/land cover or year-long surface cover of ice/snow generally more than 25% of total cover

We included quadratic effects for elevation, slope, NDVI, and solar radiation during model selection, believing that golden eagles may prefer moderate values rather than higher or lower values of these covariates. However, following standard modeling techniques, the linear term had to be present in our final model for the quadratic term to remain in the model. We conducted a Pearson’s pairwise correlation analysis prior to modeling to identify potential multicollinearity issues. We did not allow two highly correlated (|*r*| > 0.6) covariates to both be in the final model. We selected one correlated covariate over another based on perceived biological relevance. Although a pairwise correlation analysis prior to modeling is a useful tool to identify potential multicollinearity issues prior to analysis, smaller pairwise correlations can affect model estimates, and the method cannot identify scenarios where the linear combination of 2 or more model covariates are correlated to another covariate. Major changes in estimates of coefficients (e.g., negative to positive) and SEs (small to large) provide direct evidence of multicollinearity issues. Thus, we monitored estimates of coefficients and SEs during the model building procedure to ensure that multicollinearity was not influencing model estimates.

We identified covariates that might be associated with counts of golden eagles on a landscape-scale, but we did not presume to understand enough about habitat use by golden eagles to identify *a priori* candidate models to consider in an information theoretic framework [33]. We obtained a final model by backwards variable removal from the full model using the Bayesian Information Criterion (BIC [33]). The BIC often is preferred over Akaike’s Information Criterion (AIC) for finding a parsimonious model when the sample size is large and there are many covariates under consideration because the method is generally more conservative [33]. We evaluated the final model for goodness-of-fit by using the sum of the deviance residuals in a Chi-square test [25]. We also investigated whether spatial correlation existed in the model residuals using Moran’s I and comparing the correlation in residuals between transects within 200-km. A high Moran’s I value (e.g., >0.20) would be indicative of a violation of independence in the residuals and model assumptions.

We bootstrapped (1000 iterations [34]) WGES survey transects to estimate confidence intervals (CIs) for coefficients in our final model. We recalculated the inflated number of golden eagle observations within each sampled 2- x 10-km block along the bootstrapped transects using random draws from $N\left({\bar{P}}_{t},SE\left({\bar{P}}_{t}\right)\right)$ (Table 1) to account for variability in our estimates of average probabilities of detection for each observation type. The central 90% of the distribution of bootstrap estimates for each coefficient was used as the 90% CI (Percentile Method [34]). We created marginal plots to show the relationship between each covariate and the predicted relative probability of use while holding other model covariates constant at their median values.

We evaluated the performance of our habitat use model by using data from surveys conducted along the same transects in 2012 and 2013. We followed the validation process recommended by Johnson et al. [35]:

1. Predict intensity of use across sampling units and reclassify the units into 10 equal-area rank bins, e.g., bin 10 contained the highest predicted 10% of the study area.
2. Determine the median prediction *w*(*x*_*i*_) for each bin *i*.
3. Determine the utilization *U*(*x*_*i*_) value for each bin *i* using the formula
  $$U\left(x_{i}\right)=w\left(x_{i}\right)/\sum_{k=1}^{10}w\left(x_{k}\right).$$ (2)
4. Sum the count of golden eagles observed, adjusted for probability of detection, for each year separately, that fall within in each bin.
5. Estimate the expected sum of golden eagles observed, adjusted for probability of detection, within each bin for each year *j*, using
  $$N_{ij}=N_{.j}\times U\left(x_{i}\right),$$ (3)
  where *N*_.*j*_ is the total number of golden eagles observed in year *j*, adjusted for probabilities of detection.
6. Compare expected (from step 5) to observed (from step 4), using linear regression and Spearman’s rank correlation analysis.

Expected and observed values were combined among years for the regression analysis. A regression line with an intercept of 0.0 and a slope of 1.0 would indicate that predictions were proportional to observed golden eagle density. We evaluated whether slope of the regression line differed from 1.0 or if the intercept differed from 0.0 by using 90% CIs (α = 0.10). Spearman rank correlation (*r*_*s*_) coefficients were calculated for each year separately.

We mapped predictions from our model on a 2- × 10- km grid that covered the study area. We assigned each grid cell a prediction value of 1 (low use) to 10 (high use) based on the percentiles of the distribution of predictions across the study area. Prediction classes 1–10 represented the same amount of area (279,954 km^2^) on the landscape. All statistical analyses were performed in the R software [36]. We estimated the habitat use model using the **glm.nb** function based on the NB2 formulation of the negative binomial distribution available in the package MASS [37], and we performed backwards variable reduction using the **step** function available in the same package.

## Results

Sampling resulted in 1,845 2- × 10-km units that were entirely within transect lines. The number of years each sampling unit was surveyed in 2006–2010 varied from 2 to 5 (mean = 4.8); 87% of sampling units were surveyed all 5 years and 95% were surveyed at least 4 of the 5 years. The total count of golden eagles within sampling units, adjusted for probability of detection, was 1,165.

We found slope to be correlated with ruggedness (*r* = 0.63) and proportion of forest (*r* = 0.61), and thus did not include slope in the full model. Proportion of forest also was highly correlated with NDVI (*r* = 0.73). However, we were concerned about omitting either proportion of forest or NDVI from the full model so we used univariate models to compare the strength of the relationship between each covariate and counts of golden eagles. The univariate model containing proportion of forest had a lower AIC value (3039) compared to the model with NDVI (3072), so NDVI was not included in the full model. We used AIC instead of BIC in this evaluation because we were not concerned with overfitting. We also omitted the proportion of water/ice/snow from model selection because it only represented 0.4% of the landscape.

**Fig 3:**
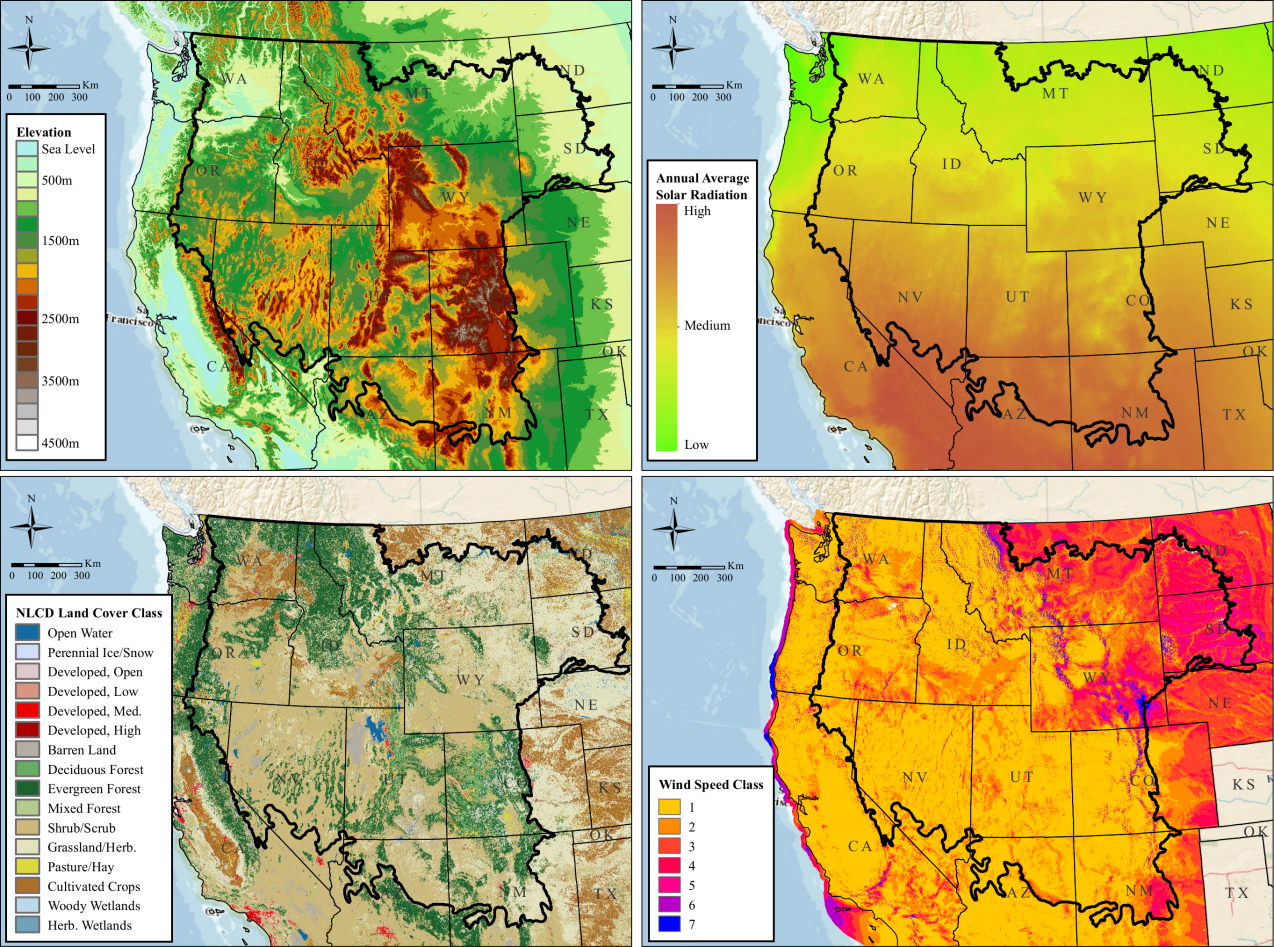
Maps depicting the four covariates in the final resource selection function. The final model contained covariates representing the median elevation (top left), median annual average solar radiation (top right), proportion of forested and developed land (bottom left), and median wind speed class (bottom right) in each sampled 2-km x 10-km rectangular cell within the study area. Image background is from The National Map (United States Geological Survey).

The full model had a BIC value of 3916, and the final model (BIC = 3802; *χ*^2^ = 1763, df = 1837, *p* = 0.89) contained covariates for elevation and elevation^2^, solar radiation and solar radiation^2^, wind speed class, and proportions of the landscape classified as forest and as developed (Table 4). Thus, the delta value [32] between these two models was 86. Marginal plots (Fig 2) illustrate the estimated relationship between predicted golden eagle use and each covariate (Fig 3). Based on the final model, with all other covariates held constant at their median values, intensity of use increased as median elevation increased up to 3012 m, then decreased as median elevation increased beyond this threshold value. While holding other covariates constant in the final model, predicted intensity of use increased as solar radiation increased to a maximum of 4040 watt-hours m^2^–^1^ day^-1^ and then decreased with increasing solar radiation. Moreover, intensity of use decreased with increasing proportions of forest or developed land. Finally, intensity of use increased as wind speed class increased.

**Table 4:** Estimates and upper and lower limits for 90% confidence intervals for coefficients in the final model of late-summer habitat use by golden eagles.

Coefficient	$\hat{\beta }$	Lower limit	Upper limit
Intercept	-15.87579665	-29.36227489	-3.38835683
Elevation	0.00187001	0.00041140	0.00339621
Elevation^2^	-0.00000031	-0.00000077	0.00000011
Solar radiation	0.00588779	0.00027881	0.01183022
Solar radiation^2^	-0.00000073	-0.00000139	-0.00000015
Forest	-2.79045017	-3.44563317	-2.08105332
Developed	-4.58784779	-14.04191034	0.80061638
Wind speed class	0.27364453	0.15989242	0.36644703

**Fig 4:**
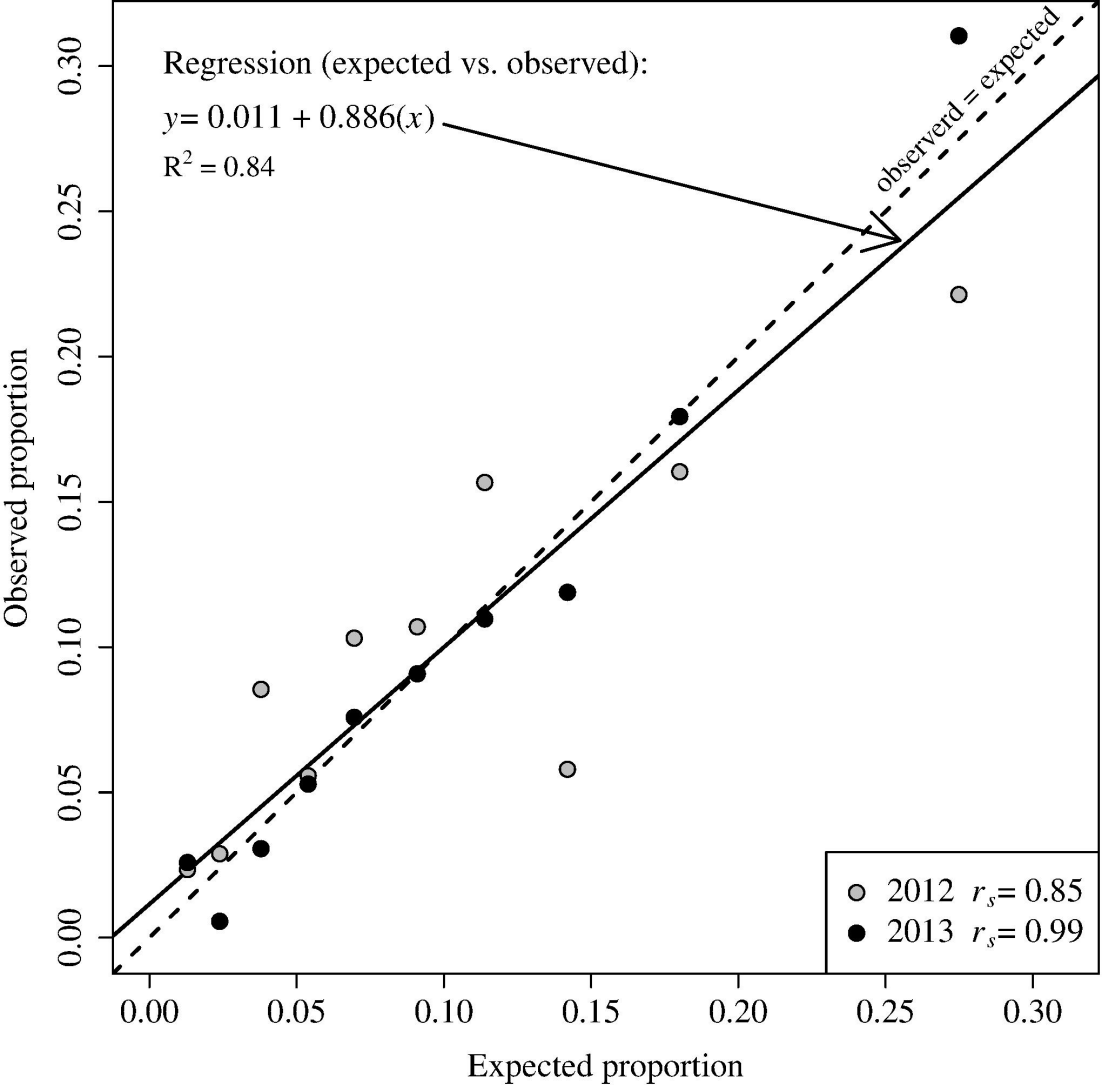
Expected versus observed proportion of golden eagle observations for the 2012 (grey circles) and 2013 (black circles) surveys in 10 habitat use bins. A perfect relationship between expected and observed use would occur along a line with an intercept of 0 and a slope of 1 (dashed line). The fitted relationship between observed and expected is shown as a solid line, and results of a Spearman’s rank correlation analyses (*r*_*s*_) are provided for each year.

There was no evidence of spatial correlation in the residuals for transects within 200-km of each other (Moran’s I = 0.03). Model validation indicated a near one-to-one relationship between observed and expected intensity of use (Fig 4). Ninety percent CIs for the intercept (-0.008 to 0.031) and slope (0.731 to 1.041) included 0.0 and 1.0, respectively. Spearman rank correlation coefficients for observed and expected intensity of use were 0.85 and 0.99 for 2012 and 2013, respectively.

**Fig 5:**
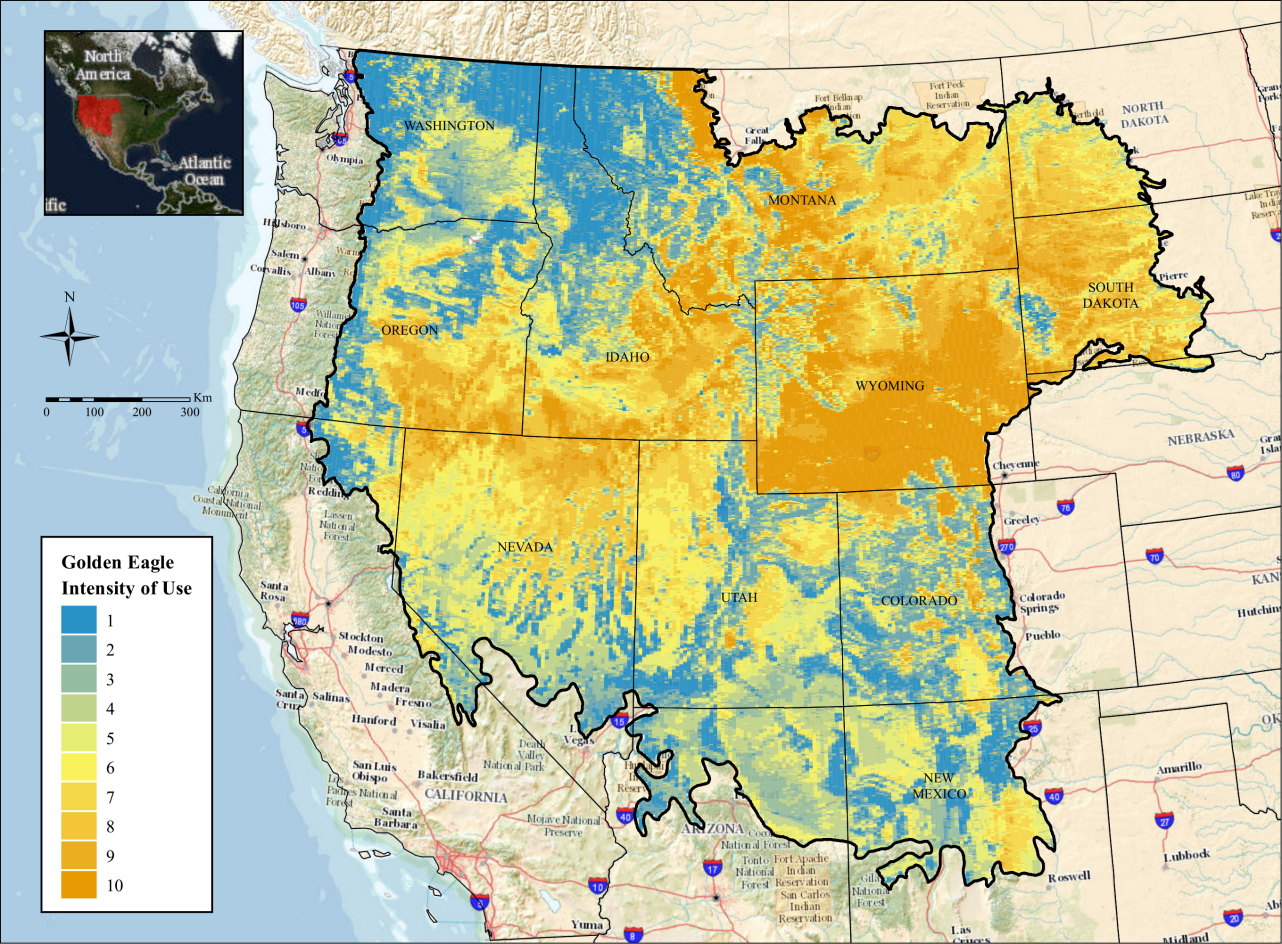
Predicted intensity of use by golden eagles in the western United States during late-summer. Image background is from The National Map (United States Geological Survey).

Based on the final model, predicted intensity of use by golden eagles during late-summer varied markedly across the study area, with lower intensities of use predicted for northwestern and southeastern portions of the study area (Fig 5). The largest contiguous area of high intensity use based on the model encompassed most of Wyoming, with smaller regions of high intensity use in central Montana, western South Dakota, southern Idaho, northern Nevada, and southeastern Oregon.

## Discussion

Ours is the first effort to model intensity of use by golden eagles across most of the species’ range in the western USA by using land cover and other environmental attributes. The results provide important insights into factors influencing golden eagle distribution across the four BCRs we surveyed in late summer. A key contribution of our model is that it did not focus narrowly on resource selection by breeding pairs of eagles, as done on local scales in other studies, but was based on all eagles detected without regard to age class or breeding status. Still, because our model framework was based on surveys of golden eagles in late summer, many observed individuals likely were tied to their respective breeding territories or natal areas. Our sample of locations of golden eagle use potentially included successfully breeding adults still on their breeding territories along with adults that failed in breeding attempts, though some of the latter may have abandoned territories by late summer [34]. Also included were recently fledged juveniles likely still on natal areas and subadult eagles, some of which also may have been associated with natal areas especially because surveys were conducted before fall migration. We acknowledge the eagle’s habitat preferences likely differ elsewhere and during other times of year.

### Habitat Use

Because our model is the first of its kind for golden eagles in the western USA, there are some limitations in comparing our findings with those of prior studies. In addition to differences in age classes and breeding status of the individuals sampled, contrasts in scale limit comparisons, as habitat use (resource selection) is scale-dependent [16]. Most studies of resource selection by golden eagles have been at second- and third-order scales, typically by comparing selection of breeding territories or of resources within territories to random points (i.e., comparing use with availability [16]). Our work was limited to a coarse scale between first- and second-order, first-order being the scale of a species’ geographic range. The effect of our scale seemed most pronounced in the influence of ruggedness, which golden eagles often select for, at least when choosing breeding territories [11,38,39]. We suspect its effect was diluted because our 20-km^2^ sampling unit size was within the 20–33 km^2^ range of average size of breeding season home ranges of golden eagles in Wyoming, northern Utah, and southwestern Idaho (summarized in Kochert et al. [5]), across the center of our study area. Thus, sampling units that included rugged features and where golden eagles were detected likely were so large that other, less rugged cover types also occurred.

Differences in definitions of covariates further obscure comparisons between our study and others. For example, scrub/shrub land cover and grassland/herbaceous land cover as defined by NLCD were omitted during backwise variable removal. This was unexpected, given the close association reported by others between breeding golden eagles and big sagebrush (*Artemisia tridentata*) shrub-steppe in much of the western USA, preference by the species for open landscapes in general [5]. For example, big sagebrush-grassland types were important to golden eagles in southwestern and south-central Montana, based on selection of breeding territories [38,39] and resources within territories [39]. In southwestern Idaho, breeding golden eagles strongly selected shrub types especially a big sagebrush-green rabbitbrush (*Ericameria nauseosa*) association [40]; grassland was a dominant land cover in home ranges of the eagles, but was used less than expected based on its availability and thus was considered “avoided.” Sagebrush shrub-steppe was an overwhelmingly good predictor of golden eagle nest site locations in Wyoming [14]. Perhaps scrub/shrub and grassland/herbaceous types did not help explain use by eagles in our study because classification of these types by NLDC was too coarse to distinguish native sagebrush and native grassland. However, a strong negative relationship between use and extent of forest cover indirectly implied that golden eagles in our study area selected for open landscapes in general. Perhaps we also found no evidence of higher use of scrub/shrub and grassland/herbaceous types because they were common across most of our study area. Regardless, we expound here because big sagebrush shrub-steppe is vital habitat for golden eagles in the western USA and its widespread, ongoing decline [9,41] is a major conservation concern for the species [5].

Intensity of use of landscapes by golden eagles we studied bore strong positive relationships with elevation, wind speed class, and solar radiation. Use increased with elevation to a threshold of 3012 m, although we did not survey areas above 3048 m due to safety concerns and access limitations. High elevation areas surveyed across our study area were coniferous forest, montane meadow, steep rock and talus, or a mix of these at the upper tree line edge. We believe golden eagles were attracted to higher elevations due to presence of optimal foraging opportunities in open areas, e.g., for yellow-bellied marmots (*Marmota flaviventris*) in meadows and talus slopes [42]. At some of these sites, strong winds may have prevailed, providing orographic updraft that favors efficient flight by eagles [43,44], although we did not broadly detect correlation between elevation and wind speed class. Last, golden eagles may use higher elevations to mitigate high summer temperatures, especially in southern parts of their western USA range [45].

Evidence in our model that use of landscapes by golden eagles decreased with increased extent of developed area was unsurprising. The species typically avoids such areas and may abandon traditional nesting territories areas when development encroaches [6,10]. Lack of importance of the crop/hay land cover type in our model was difficult to place in the context of what is known about second- and third-order resource selection by golden eagles in the western USA, at least by breeding pairs. Agricultural lands generally are avoided [5,38], although perennial herbaceous haylands, a subset of agricultural lands, could possibly attract prairie dogs (*Cynomys* spp.) and thus golden eagles. Fine-scale distinction of agricultural land cover types may lend more insight on selection by golden eagles, albeit at higher levels of selection.

Use intensity by golden eagles increased with increasing solar radiation to a value of 4040 watt-hours m^2^–^1^ day^-1^ then decreased. This maximum level corresponds to an east-west zone extending roughly to the northern one-third of our study area (Fig 3). Open, more arid landscapes south of this zone may support lower densities of golden eagles because lower primary productivity associated with increased aridity probably supports a lower average abundance of small herbivore prey [46,47].

To summarize our findings on habitat use, our data suggest late-summer densities of golden eagles within most of the interior western USA tend to be greatest in open, relatively undisturbed landscapes with high wind potential. The densities also are positively influenced by elevation and moderate levels of solar radiation. Conversely, densities decline with increasing solar radiation and aridity, perhaps due in part to associated decreases in densities of prey. A few unexpected findings, e.g., no evidence of preference for scrub/shrub and grassland/herbaceous types, may in part be due to obfuscation of local habitat relationships by the large spatial scale of our study approach.

### Predicted Intensity of Use across the Western USA

Based on our model, the largest contiguous area of high intensity use by golden eagles in the four BCRs we sampled in late summer was predicted to be in Wyoming, with smaller regions of high intensity use in central Montana, western South Dakota, southern Idaho, northern Nevada, and southeastern Oregon. This pattern seems supported, in general, by what is known about abundance of breeding golden eagles in parts of the western USA. For example, Wyoming, USA is known to have high breeding densities across most of the state; 12 survey areas covering 8% of Wyoming supported a mean of one breeding pair per 60 km^2^ [38]. An average of one breeding pair per 139 km^2^ occurred in major mountain ranges (R. Oakleaf, in Phillips et al. [48]), where more moderate intensity of use was indicated by our predictive map for Wyoming.

Golden eagle density was estimated annually in 2006–2010, 2012, and 2013 for each BCR making up the area covered by the WGES [2,49]. Using the relative density map presented in this paper, we could estimate golden eagle densities for regions within the larger study area that do not necessarily adhere to BCR boundaries. For example, we can estimate golden eagle density during late-summer in the portion of Wyoming, USA that overlaps both BCRs 16 and 17. This can be done by scaling the predictive map for the entire study area to sum to the most recent abundance estimate for the entire study area (29,757 golden eagles [49]), and then summing the predictions among sampling units with center points falling within the state’s boundaries. Obtaining measures of precision for such estimators requires a multi-level bootstrap similar to the one we described above that would include both variability in the estimated population total and variability in the prediction map.

We emphasize that our landscape-scale model is intended for use across larger geographic regions (i.e., closer to first-order resource selection), and does not accurately represent habitat use within home ranges (i.e., third-order selection) or local projects such as wind energy facilities. Our choice of sampling unit size (2 × 10 km) was driven by the original survey design and not golden eagle biology. We considered using smaller sampling units, but computational costs due to the size of the study area prohibited this. To improve the model, we can augment the underlying data with additional surveys, refine covariates such as some land cover types, and consider additional covariates. Model validation with data not used for modeling indicated predictions of use along survey transects were quite accurate. We recognize that some individual, resident eagles seen in 2006–2010 may have been observed along the respective transects in 2012–2013, but juveniles and non-resident eagles also were represented in the validation data and we considered the samples sufficiently independent. In the future, we also could use data from golden eagles marked with telemetry devices to help validate our predictions; multiple broad-scale studies currently are underway or are being initiated in the western USA (U.S. Fish and Wildlife Service, Division of Migratory Birds, Washington, DC).

### Management Implications

In 2013, the Service initiated a “systematic conservation planning framework” for golden eagles in the western USA, consisting of “structured, spatially explicit assessments of risk and conservation opportunity within and across unique landscapes… mainly to address concerns with impacts of energy development.” (U.S. Fish and Wildlife Service, Division of Migratory Birds, Washington, D.C.). This includes desk-top assessments to help prioritize landscapes for various conservation and mitigation efforts and prompt closer evaluations. Our model can help support this and similar planning endeavors by complementing spatial information on migration corridors, major overwintering sites, and important breeding areas of golden eagles. Moreover, our predictive map can be part of a suite of tools energy developers can use to screen landscapes for potential risk to golden eagles. Last, our model could help support refined estimates of the size of local populations of golden eagles, critical to the Service’s decisions on levels of take of golden eagles annually authorized by permit [12]. Golden eagles within 224 km of a potential source of impact comprise a “local-area population” [13]. Currently, the Service bases its estimate of the size of such a population on an assumption that golden eagle density within the area is uniform and equals that estimated for the entire BCR that encompasses it (if the local-area overlaps more than one BCR, densities for each zone of overlap are assumed to equal those of the respective BCRs [10]). Based on our model and refinements thereof, population size could be estimated for habitat subunits within a local-area then these estimates could be compiled to more plausibly approximate local-area population size.
